# Symptoms assessment and decision to treat patients with advanced Parkinson’s disease based on wearables data

**DOI:** 10.1038/s41531-023-00489-x

**Published:** 2023-03-27

**Authors:** Clara Virbel-Fleischman, Flavien Mousin, Shuo Liu, Sébastien Hardy, Jean-Christophe Corvol, Isabelle Benatru, David Bendetowicz, Matthieu Béreau, Valérie Cochen De Cock, Sophie Drapier, Solène Frismand, Caroline Giordana, David Devos, Yann Rétory, David Grabli

**Affiliations:** 1 Sorbonne University, Brain Institute – ICM, Inserm, CNRS, Paris, France; 2 Centre EXPLOR!, Air Liquide Healthcare, Gentilly, France; 3 APHP, Pitié-Salpêtrière Hospital, Neurology Department, Paris, France; 4 Poitiers University Hospital, Neurology Department, Poitiers, France; 5 INSERM, Poitiers CHU, Poitiers University, Centre d’Investigation Clinique CIC, 1402 Poitiers, France; 6 Besançon CHRU, Neurology Department, Besançon, France; 7 Beau Soleil Clinic, Sleep et Neurology Department, Montpellier, France; 8 EuroMov Digital Health in Motion, Montpellier University, IMT Mines Ales, Montpellier, France; 9 Rennes CHU, Neurology Department, Rennes, France; 10 Nancy CHRU, Neurology Department, Nancy, France; 11 Nice University Hospital, Neurology Department, Nice, France; 12 Lille University, Lille CHU, Inserm, U1172, Lille Neuroscience & Cognition, NS-park F-CRIN network, LICEND, Lille, France; 13 Paris-Saclay University, Laboratoire Complexité, innovations, activités motrices et sportives (CIAMS), 91405 Orsay, France; 14 Orléans University, CIAMS, 45067 Orléans, France

**Keywords:** Parkinson's disease, Health care

## Abstract

Body-worn sensors (BWS) could provide valuable information in the management of Parkinson’s disease and support therapeutic decisions based on objective monitoring. To study this pivotal step and better understand how relevant information is extracted from BWS results and translated into treatment adaptation, eight neurologists examined eight virtual cases composed of basic patient profiles and their BWS monitoring results. Sixty-four interpretations of monitoring results and the subsequent therapeutic decisions were collected. Relationship between interrater agreements in the BWS reading and the severity of symptoms were analyzed via correlation studies. Logistic regression was used to identify associations between the BWS parameters and suggested treatment modifications. Interrater agreements were high and significantly associated with the BWS scores. Summarized BWS scores reflecting bradykinesia, dyskinesia, and tremor predicted the direction of treatment modifications. Our results suggest that monitoring information is robustly linked to treatment adaptation and pave the way to loop systems able to automatically propose treatment modifications from BWS recordings information.

## Introduction

Treatment of Parkinson’s disease (PD) is mainly based on dopaminergic substitution pharmacotherapy. However, the response to dopaminergic drugs evolves throughout the disease course. After 7–10 years from onset, fluctuations and dyskinesia eventually occur^1^. At this stage, patients may require device-aided therapy (DAT) to overcome the challenges of treatment optimization. All DAT, including deep brain stimulation, continuous subcutaneous apomorphine infusion (CSAI) or levodopa-carbidopa intestinal gel are efficient in reducing fluctuations and improving quality of life^2–4^. However, making the right decision regarding DAT initiation or follow-up may be difficult, partly because collecting information about symptoms and their daily variations is challenging^5^. Objective measurement of movements may increase the level of accuracy in symptom evaluation and better guide therapeutic decisions^6^. Body-Worn Sensors (BWS) are devices that continuously monitor activity during daily-life without medical supervision, and translate motor parameters into normal movement, akinesia, dyskinesia or tremor. BWS monitoring use is spreading^7,8^ and expert panels have investigated usage to provide guidance for application in clinical practice^9^.

The PKG System (Global Kinetics Corporation ®, Australia) is a wrist-worn device that automatically quantifies bradykinesia, dyskinesia and tremor. The PKG algorithm has been validated against current standards of evaluations in PD: experts assessment^10,11^, video^12^, patient diaries^13^, and clinical rating scales^14^. There is emerging evidence suggesting that it could be a valuable tool for therapeutic management^7,9,15–17^. However, this requires further confirmation. Importantly, how information from a BWS, including PKG, is translated into symptoms by clinicians in order to identify relevant targets for treatment adaptation is still a black box. Thus, our aim was to (i) highlight the relevant outcomes of PKG and reveal if this information is consistently identified by the clinicians, (ii) examine therapeutic decisions based on this information in advanced patients with apomorphine pump, and (iii) investigate the link between outcomes and decisions.

## Results

### PKG reading

The interrater agreements in the evaluation of bradykinesia, dyskinesia and tremor for all cases was high (median in % [range in %]: 89 [72, 100], 93 [71, 100] and 99 [89, 100], respectively) (Supplementary Table 1).

**Fig. 1:**
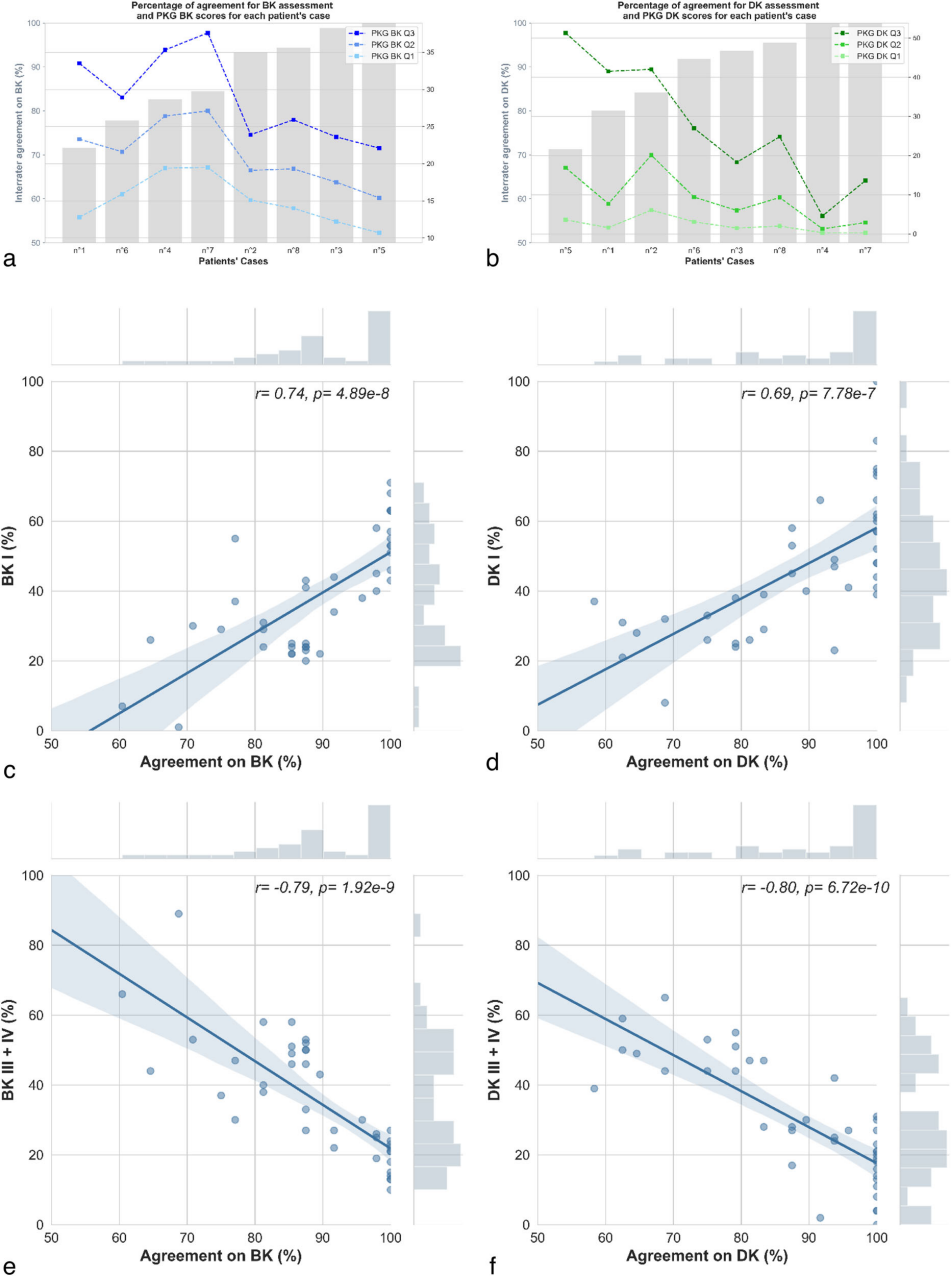
PKG bradykinesia and dyskinesia reading. **a**, **b** Percentage of interrater agreement for each patient’s case in bradykinesia and dyskinesia assessment (bars in gray, left Y-axis) and global PKG BKS and DKS as quartiles and median (blue dots for bradykinesia and green dots for dyskinesia, right Y-axis). Patient cases were sorted by agreement in the symptom assessment by raters. **c**, **d** Representation of the correlation between the interrater agreement in bradykinesia and dyskinesia assessment and the percent of time spent by periods of 3 h in the lowest level of severity of bradykinesia and dyskinesia, respectively (BK I and DK I). *r*: correlation coefficient and *p* value. **e**, **f** Representation of the correlation between the interrater agreement in bradykinesia and dyskinesia assessment and the percent of time spent by periods of 3 h in the highest levels of severity of bradykinesia and dyskinesia, respectively (BK III + IV and DK III + IV). *r*: correlation coefficient and *p*: significance from the Spearman correlation. In **c**, **d**, **e** and **f**, the dots represent the percentage of agreement in the assessment of the symptom for each percent of time spent in the severity level of the symptom. The fill-lines represent the correlation with confidence intervals in transparency.

The association between interrater agreements in the symptom assessments and the severity of bradykinesia and dyskinesia PKG scores expressed at different time scales is described in Fig. 1a, b respectively. The agreement in bradykinesia assessment was negatively correlated with the integrated PKG scores reflecting bradykinesia’s highest severities (BK Q2: *r* = −0.73, *p* = 0.04, and BK Q3: *r* = −0.76, *p* = 0.03 (global second and third quartiles of the bradykinesia score); n = 8). There was no correlation with either the global first quartile of the bradykinesia score (BK Q1) or the Percent of Time Immobile (PTI). The agreement in bradykinesia assessment was negatively associated with the continuous measures of bradykinesia (BKmed (median of the continuous bradykinesia score): *r* = -0.58 *p* < 0.0001, and BK75 (third quartile of the continuous bradykinesia score): *r* = −0.54, *p* < 0.0001; *n* = 272).

The agreement in dyskinesia assessment was negatively correlated with the integrated PKG scores reflecting dyskinesia’s highest severities (DK Q2: *r* = −0.75, *p* = 0.03, and DK Q3: *r* = −0.95, *p* = 0.0003 (global second and third quartiles of the dyskinesia score); *n* = 8). There was no correlation with the global first quartile of the dyskinesia score (DK Q1). In addition, the interrater agreement in dyskinesia assessment was negatively correlated with the continuous measures of dyskinesia (DKmed (median of the continuous dyskinesia score): *r* = −0.63, *p* < 0.001, and DK75 (third quartile of the continuous dyskinesia score): *r* = −0.60, *p* < 0.0001; *n* = 272), and with the Fluctuation Dyskinesia Score (FDS: *r* = -0.90, *p* = 0.003; *n* = 8).

The interrater agreements were also significantly associated (*p* < 0.0001) with the percentages of time spent in severity levels I and III + IV by intervals of three hours (*n* = 40, Fig. 1). For bradykinesia and dyskinesia, the association was positive for the lowest severity level (*r* = 0.74 (Fig. 1c) and *r* = 0.69 (Fig. 1d), respectively), while it was negative for the highest severity level (*r* = -0.79 (Fig. 1e) and *r* = −0.80 (Fig. 1f), respectively).

Interrater agreement was also studied in line with variations in symptoms across the monitoring period. Interrater agreements for bradykinesia and dyskinesia were not correlated with the coefficients of variation of the continuous bradykinesia scores (BKmed nor BK75) and of the continuous dyskinesia scores (DKmed nor DK75), respectively.

**Fig. 2:**
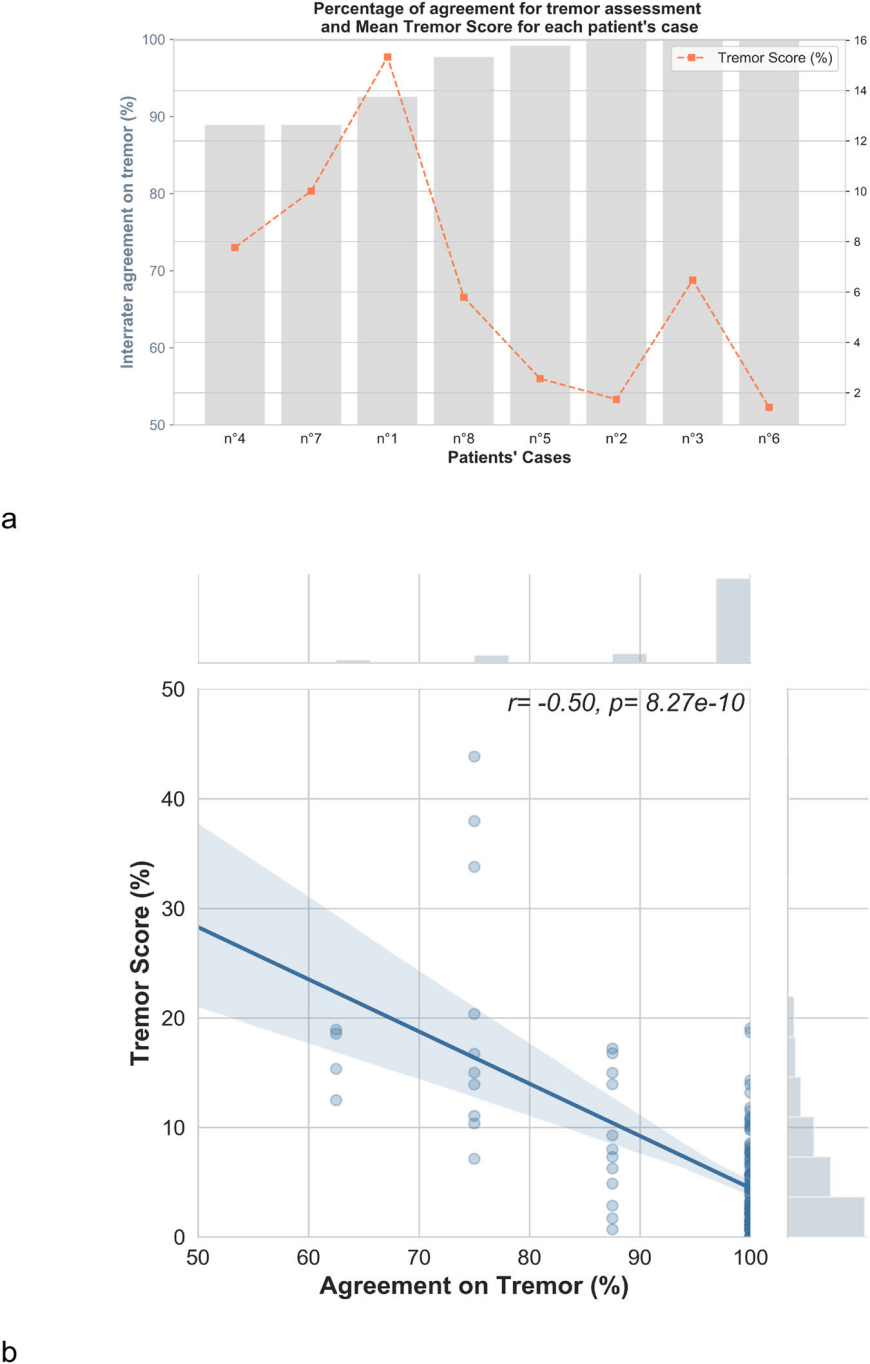
PKG tremor reading. **a** Percentage of interrater agreement for each patient’s case in tremor assessment (bars in gray, left Y-axis) and Tremor Score (orange dots, right Y-axis). Patient cases were sorted by agreement in the symptom assessment by raters. **b** Representation of the correlation between the interrater agreement in tremor assessment and the Tremor Score. The dots represent the percentage of agreement in the assessment of the symptom for each percent of time spent in the severity level of the symptom. The fill-line represents the correlation with confidence intervals in transparency. r: correlation coefficient and p: significance from the Spearman correlation.

Moreover, correlation studies showed that interrater agreement in tremor assessment was negatively associated with the Tremor Score (*r* = −0.50, *p* < 0.0001, *n* = 136; Fig. 2), and with the Percent of Time with Tremor (PTT: *r* = -0.94, *p* < 0.001; *n* = 8). Finally, there was no relationship between the agreement for tremor and the intra-case variability of the Tremor Score.

### Treatment adjustments

**Fig. 3:**
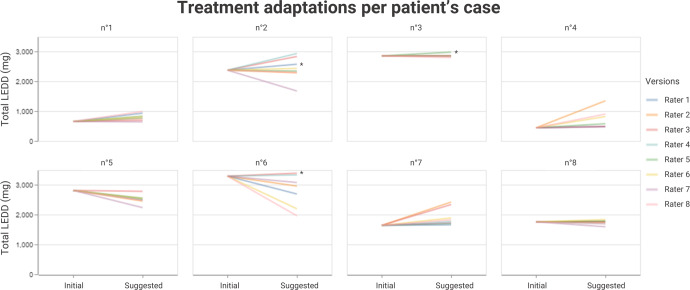
Treatment adaptations per patient case suggested by all raters. One color is attributed to each rater. Evolution between the initial total LEDD (Levodopa Equivalent Daily Dose) and the eight suggested LEDD are presented for each case. Augmentations of total LEDD suggested by Rater 1 for Case no. 2, Rater 5 for Case no. 3, and Rater 3 for Case no. 6 were only based on the addition of Amantadine, which corresponds to a therapeutic action towards the reduction of antiparkinsonian treatment. They are noted by the asterisk sign (*).

The raters suggested modifications of the initial treatments for all patients such that the total LEDD (Levodopa Equivalent Daily Doses) could change and be different for the same patient case. The total LEDD suggested by the adaptations ranged from 60% to 305% of the initial treatments (Fig. 3). Total LEDD augmentations suggested in Cases no. 2, no. 3 and no. 6 by Rater 1, Rater 5, and Rater 3, respectively, were exclusively due to amantadine addition in order to reduce dyskinesia and could thus be considered in agreement with dose reduction for other dopaminergic medications. The agreement in treatment direction was globally high (median in % [range in %]: 81 [50, 100]; Supplementary Table 1), but varied among the cases. The raters unanimously agreed for two cases (no. 5 and no. 7) and disagreed at 50% for one (no. 8).

### Link between the case analysis and treatment adjustments

Finally, we tried to better understand the relationship between the treatment modifications and information from the patients and their PKG. We could not establish any significant correlation between the agreement in treatment modification and the agreement in the assessment of the three symptoms.

Thus, we studied independently the 29 suggestions of total LEDD increase, the eight suggestions for stabilization and the 27 decrease suggestions. The resulting model had good accuracy (classification rate of 86%).

Whatever the patient characteristics nor the PKG results, each individual raters had different tendencies in suggesting treatment modifications for each patient. Indeed, the fact of being a specific rater had an impact on the decision for the treatment increase, stabilization, or decrease (Supplementary Table 2). Also, the case variable (*i.e*. the fact of being a case without considering the other variables) had an impact on the treatment modification direction, independently from age, initial total LEDD and FDS, PTT and PTI (Supplementary Table 2).

From a clinical point of view, age, initial total LEDD, FDS, PTI and PTT could predict the LEDD change. The lowest initial total LEDD favored an increase of the total LEDD. The highest PTI and PTT scores favored an increase of total LEDD while the highest FDS were in favor of its decrease (Table 1). Reciprocally, the opposite values favored the reverse action. In comparison to the other parameters, age had no or less impact (coefficient’s absolute mean value below 0.3, Table 1).

**Table 1:** Impacts of the patients and PKG information on the treatment modification: increase, stabilization or decrease of total LEDD.

	Increase (*n* = 29) Mean (ic90%)	Stabilization (*n* = 8) Mean (ic90%)	Decrease (*n* = 27) Mean (ic90%)
Age	−0.289 (−0.374, −0.210)	−0.099 (−0.183, −0.027)	−0.199 (−0.290, −0.110)
FDS	−0.320 (−0.434, −0.193)	−0.861 (−0.933, −0.788)	0.398 (0.269, 0.510)
PTI	1.011 (0.912, 1.104)	−1.140 (−1.211, −1.064)	−0.590 (−0.708, −0.480)
PTT	1.158 (1.036, 1.302)	−0.232 (−0.330, −0.130)	−1.428 (−1.562, −1.306)
Initial total LEDD	−1.182 (−1.327, −1.076)	0.154 (0.049, 0.261)	0.732 (0.600, 0.871)

Mean: estimated effect of the factor from the 1000 iterative resamplings at 80% of the 64 combinations.

*ic90%* credible interval at 90% from the distribution of the bootstrapping distribution.

## Discussion

Although not unanimous, the PKG reading for the assessment of bradykinesia, dyskinesia and tremor, and the directions of treatment adaptations were consistent among neurologists for each case. Scores that summarize these symptoms in the PKG could influence the decision: the highest FDS scores favored a total LEDD decrease, and the highest scores of PTT and PTI favored its increase.

The variability in scores describing the symptoms could be a reason for the discrepancy between neurologists. Actually, the agreements in bradykinesia, dyskinesia and tremor assessments were not associated with the intra-case variability of severity, but directly with the symptoms’ severity. The most severe PKG symptoms resulted in the lowest agreement. Moreover, the highest symptom severities generally go along with the highest number of events, which engender more detections of events by the neurologists, and as such, a higher likelihood of discrepancy resulting in less agreement. Our results highlight the need for standardizing the reading of monitoring results, especially for the most severe cases, which necessarily need treatment adaptations.

There was no consensus in the direction of the treatment modifications for each patient, even if on average more than 6 out of the 8 neurologists agreed on the therapy direction of the case. Either discrepancy in the PKG reading could not allow for a unique decision, or the information collected was not sufficient. In the trial by Santiago et al., the four study neurologists found that monitoring results did not provide useful information in addition to the visit for 59% of the cases^15^. The disparity in the neurologists’ previous experience of the PKG might explain the differences in readings of the results, and thus, decisions on a treatment action^18^. Perhaps also, the type of presentation of results may influence the interpretation of the monitoring results. For example, a binary display of PD symptoms, such as dichotomous ON and OFF states, may not lead to a wide variability of interpretation^19^. Our study highlights the need for explicit criteria as the heterogeneity of interpretation might modify decisions in therapeutic actions. Moreover, this heterogeneity triggers the question of reliability when controlled trials involve the interpretation of monitoring results.

Finally, we found a consistent relationship between the integrated data of the PKG (FDS, PTT and PTI) and the dominant therapeutic decisions. As we especially studied reports of patients treated with Apomorphine, we explored key elements that may be involved in the automatization of DAT adaptations. Pahwa et al. suggested that target scores should help neurologists to better integrate BWS into clinical practice^8^, which may support the adoption of such technology^20^. Precisely, Farzanehfar et al. provided guidance to neurologists through the definition of targets in PKG, especially the Percent of Time Over Target which can be provided by PKG, but was not available in our study. They found that monitoring results could influence the therapeutic decision in 61% of cases^7^. Another factor that could account for the discrepancies in treatment adaptation agreement was the lack of explicit recommendation to follow published guidelines regarding treatment adaptation in patients with motor complications. Usually, the treating neurologist knows the patient, and no decision depends only on a “virtual” case. The artificial situation that we created might exist in the future with intelligent systems that can directly identify candidates for DAT^21^, predict the response to treatment^16^, or regulate its delivery^22^. A first approach may be an intelligent system that triggers alerts when selected parameters exceed any determined threshold based on BWS monitoring and suggests an intervention by specialists. Such a hybrid system leaves the full decision to the specialists and the possibility of having access to other parameters that the patient would report best. Another more ambitious and innovative approach is a closed-loop system that would allow online adaptations of dopaminergic treatments, for example the apomorphine flow rate, based on continuous measurement of motor parameters from a BWS. A first attempt was performed by Rodriguez-Molinero et al. studying the real-time impact of variations of the apomorphine flow rate on the results of a BWS monitoring^22^. The system showed some tendencies indicating improved symptom control and thus is an incentive for further development of closed-loop techniques.

To assist in therapeutic decision making, our work suggests an original approach by selecting relevant parameters from among the information available from a single BWS. Among the strengths of this study, the patient cases represented a variety of situations encountered in PD. According to the PKG instructions, they were described with no or uncontrolled fluctuations, with or without tremor, and close to subjects without PD. In addition, as the goal of our study was to provide latitude for maneuvering and learning about PKG reading in real-life, we purposefully gave no precise instructions to the neurologists, which could have reduced variability in event detection.

Some pitfalls should be considered. First, clinical studies with a larger sample of neurologists and decisions regarding treatment adaptation are needed to further develop the process of criteria selection and the development of automated systems for treatment adaptation. Second, the neurologists’ readings might not be entirely replicable due to variability in PKG expertise. Moreover, as they did not know the patients, the neurologists may have required additional information such as nonmotor symptoms and the presence of impulse control disorders that are not directly provided by a BWS monitoring. Nonetheless, a few studies showed correlations between the objective monitoring of motor symptoms and the appearance of nonmotor symptoms^23–25^. Especially the BKS and DKS (Bradykinesia Score and Dyskinesia Score, respectively) of the PKG have been associated with several nonmotor symptoms of the NMS scale by van Wamelen et al.^23^. Those exploratory results support the hypothesis that adjusting only from objective monitoring may consider both motor and nonmotor fluctuations, making possible the idea of a closed-loop system with the BWS and a DAT. Collecting this information via more elaborate systems such as connected health platforms would be another solution^26,27^.

Our findings explore an emergent area in the management of PD. We created situations where neurologists could make decisions based only on BWS reading and found that the automation of treatment adjustments will not be trivial. Although our findings can guide the selection of relevant information from objective monitoring that are related to decisions in therapy adjustments, we cannot consider the reading of monitoring results as a gold standard because of current varying displays of BWS monitoring results and discrepancies in reading. Targets in the objective measurement results may be a first guidance level to automate decisions. Standardizing the way information from BWS is analyzed may facilitate more consistent interpretations and treatment modifications by PD specialists in routine practice, and in the context of DAT counseling, it would also benefit the implementation of these intelligent systems^20^.

## Methods

We conducted an observational study among neurologists that were asked to analyze the results of PD patient monitoring. At the time of the study (August 2019 - February 2020), participants were investigators of a randomized controlled trial (@ctipark, NCT03213379) from which patients’ data was collected and used as part of this study. The patients included in the @ctipark trial gave their written consent. A local ethics committee approved this study (CPP Nord Ouest IV).

The @ctipark trial was an interventional, comparative, randomized, prospective, French study and multicentric carried out in hospitals initiating treatment with apomorphine pump, in adult patients with Parkinson’s disease. The working hypothesis is that the use of an objective monitoring procedure will allow reduce the consumption of healthcare (reduction in the number of consultations and of hospitalizations) compared to the usual care thanks to a greater accuracy of the assessment of the patient’s condition. The benefit would therefore be on the one hand a health savings and on the other hand a more precise, faster therapeutic balance and therefore more satisfying for patients.

Included patients were men or woman aged up to 75 years old with Parkinson’s disease and for whom a first-time installation of the apomorphine pump was planned and who agreed wearing the device according to the terms of the protocol.

### Procedure

**Fig. 4:**
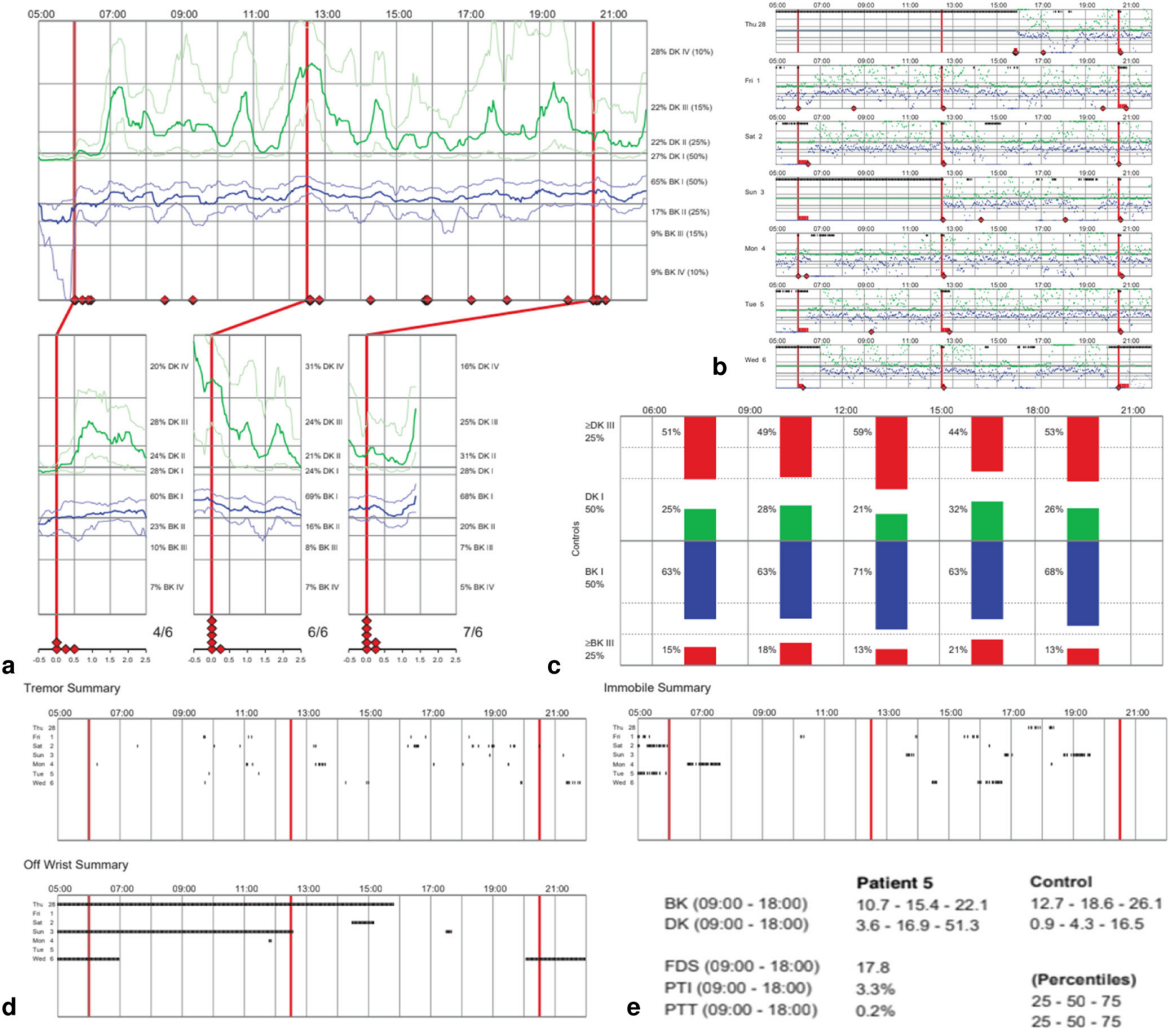
Example of a patient’s case PKG. The PKG was provided as it was collected after creation by the proprietary algorithm. **a** Main graph, from which the continuous parameters BKmed, BK75, DKmed, and DK75 were obtained via standardized methods. **b** Detailed evolution of bradykinesia and dyskinesia per 2-min periods for each day along the entire period of monitoring. **c** Second graph that displays the percentages of time at severity levels I and III + IV per periods of 3 h, and from which BK I, BK III + IV, DK I, and DK III + IV were collected. **d** Three summary graphs for tremor occurrence, time with the PKG Watch off wrist, and immobile time. The tremor summary graph was used to create the continuous percent of time with tremor (Tremor Score) via standardized methods. **e** Summarized scores displayed at the end of the PKG, from which the global first, second and third quartiles of the BKS and DKS (BK Q1, BK Q2, BK Q3, DK Q1, DK Q2, DK Q3), and the FDS, PTI and PTT were collected.

Eight neurologists (raters) considered as movement disorder specialists volunteered to participate in this study. They all practiced in Parkinson’s disease specialty centers with 3–20 years (median 13 years) of experience in neurology. The raters had been trained for PKG reading and interpretation as part of the @ctipark study with either the realization of ten PKG interpretations in their practice or the realization of four PKG interpretations and one succeeded test. They analyzed 9 [2, 50] PKG readings before their participation (median [minimum, maximum]). For all cases submitted to the raters, the patients were treated with conventional medication and CSAI. Selected information from eight random patients constituted the eight cases (Table 2):

- Context (age, gender, weight, height, duration of PD since diagnosis, duration of dyskinesia since appearance, scores of the Movement Disorder Society - Unified Parkinson’s Disease Rating Scale and Montreal Cognitive Assessment scale when available), antiparkinsonian therapy with conventional treatment and apomorphine pump, and therapies for major comorbidities, if present;
- PKG automatically generated by the proprietary algorithm, containing the graphs and the summary of scores but with no report summary that may be provided by the society (Fig. 4).

**Table 2:** Patients demography.

Patients cases	Gender	Age (yo)	Weight (kg)	Height (m)	Duration of PD (y)	Duration of dyskinesia (y)	MDS-UPDRS	MoCA	Conv. LEDD (mg)	Apo. LEDD (mg)	FDS	PTI	PTT
no. 1	M	53	92	1.78	16	NA	59	27	175	480	18.1	7.3	1.1
no. 2	F	68	41	1.55	18	8	NA	25	1420	960	14.9	1.5	0.1
no. 3	M	72	75	1.70	7	5	16	27	1360	1500	10	0.4	0.6
no. 4	M	58	78	1.68	4	2	NA	30	250	192	6.5	6	1.3
no. 5	M	53	70	1.82	11	8	NA	27	1940	880	17.8	3.3	0.2
no. 6	M	73	91	1.81	17	6	17	NA	2578.5	720	12.9	3.6	0.1
no. 7	M	64	77	1.73	7	3	24	26	993.75	650	11.2	12	1.2
no. 8	M	70	73	1.64	13	5	NA	27	983.5	780	12.2	2.8	0.5

MDS-UPDRS is the total score obtained after evaluation with the Movement Disorder Society – Unified Parkinson’s disease Rating Scale. MoCA is the total score obtained after evaluation of the patient with the Montreal Cognitive Assessment scale. Conv. LEDD is the Levodopa Equivalent Daily Dose of conventional treatment; Apo. LEDD is the Levodopa Equivalent Daily Dose of Apomorphine delivered by pump. FDS, PTI and PTT are the summarized scores obtained with the PKG.

*NA* not available.

We used an identical number of raters and subjects to minimize variance in interrater reliability^28^. All eight raters studied all eight cases resulting in 64 unique combinations to study.

### Data collection

The PKG Watch was worn over seven days for real-life monitoring at each patient’s home. The monitoring results (PKG) showed the progression of bradykinesia (BKS) and dyskinesia (DKS) during the daytime (5 a.m. to 10 p.m.) at four levels of severity (I, II, III and IV). BKS and DKS were produced every two minutes and displayed in the main PKG graph as the median and the first and third quartiles of severity scores, for all days monitored and according to time. A second graph showed the percent of time spent in severity levels I and III + IV, per interval of 3 h from 6 a.m. to 9 p.m. The PKG also displayed graphs for the occurrence of tremor, immobility periods and a summary of scores.

**Fig. 5:**
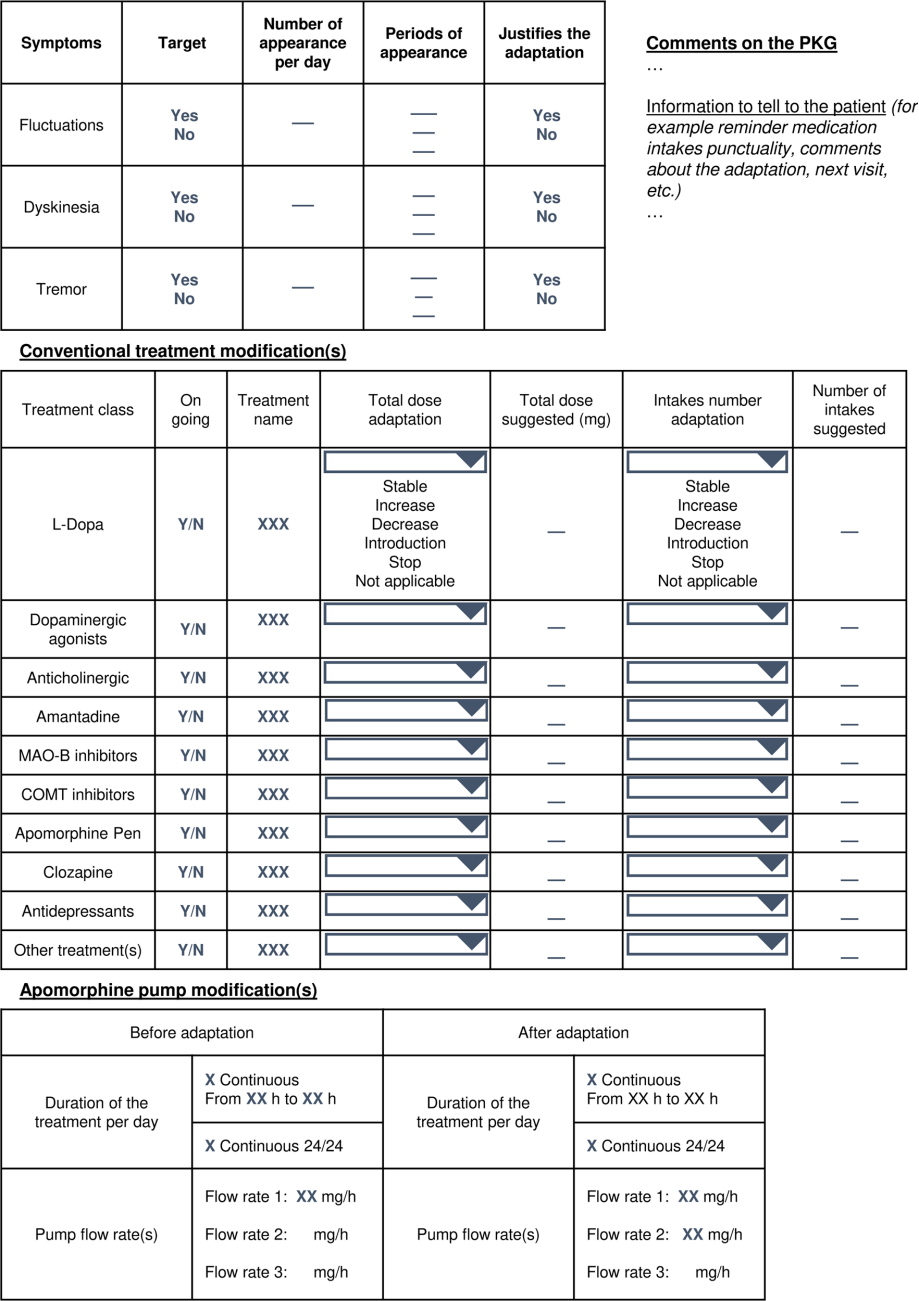
Example of a questionnaire for a patient’s case evaluation by the rater. A first table represents the reading of the PKG. The rater also has the possibility to add comments on the PKG or suggest information to tell to the patient. A second table is dedicated to the suggestions of conventional treatment modifications and a third table is related to the apomorphine pump therapy suggestions of modifications. For conventional treatments, the rater has the choice between stabilization, decrease, increase, initiation or stop of the treatment regarding doses and number of intakes per day. For apomorphine delivery, several flow rates and during different periods of delivery may be suggested.

The eight raters received a two-part questionnaire with each patient’s case to report their evaluation and suggest treatment adaptations (Fig. 5).

The first part applied to the PKG reading by raters. They had to signal if the symptom (bradykinesia, dyskinesia, and tremor) was significant, and to specify the number of occurrences per day they observed and the duration of each period.

The second part of the questionnaire was related to treatment adaptation suggestions for conventional treatment (total dose, number of intakes) and CSAI therapy (duration of apomorphine delivery and flow rates).

Integrated information directly provided by the PKG and reflecting the severity of symptoms with various time scales were collected:

- Percentages of time with bradykinesia or dyskinesia at severity levels I and III + IV, per periods of 3 h (BK I, BK III + IV, DK I, DK III + IV);
- The global first (Q1), second (Q2) and third (Q3) quartiles of the BKS and DKS (BK Q1, BK Q2, BK Q3, DK Q1, DK Q2, DK Q3);
- Summarized Fluctuation Dyskinesia Score (FDS, which is the range of the interquartile of BK and DK throughout the recording period);
- Summarized Percent of Time Immobile (PTI, which is the percentage of time the patient was motionless between 9 a.m. and 6 p.m.);
- Summarized Percent of Time with Tremor (PTT, which is the percentage of time with tremor between 9 a.m. and 6 p.m.).
  Other continuous parameters from the PKG were obtained via standardized methods:
- Continuous BKS and DKS medians and third quartiles (BKmed, BK75, DKmed, DK75, respectively): severity level interpreted by 30-minute periods by visual interpretation of the main graph from 5 a.m. to 10 p.m. From each time period, the score (I to IV) was determined from the severity level that occupied the most time.
- Continuous percent of time with tremor (Tremor Score): hourly score obtained from an image analysis of the tremor graph. Each hour was extracted separately via a transitional disclosure of the color intensity of the global frame and the representation of tremor episodes in dots. The Tremor Score was calculated using the space filled by dots per hour over the 7-day monitoring period. Contrary to the PTT which is a unique global score for the entire period of monitoring, the Tremor Score characterizes each of the 17 h monitored by the PKG during the day (from 5 a.m. to 10 p.m.).

From these continuous scores, coefficients of variation were computed per patient case, with the score means and standard deviations obtained by a 30-min period to obtain intra-case variability.

### Data analysis

The raters’ answers were gathered by patient case. The number of raters detecting a symptom at each 30-min period was computed for bradykinesia, dyskinesia and tremor from 5 a.m. to 10 p.m. The agreement between raters for each period in the assessments of bradykinesia, dyskinesia and tremor were independently computed by taking into account detections and non-detections of each symptom (range [50; 100]%).

Conventional and apomorphine Levodopa Equivalent Daily Doses (LEDD) were computed following Tomlinson et al.^29^ to obtain total LEDD (sum of the two). We studied the difference between the initial total LEDD and the total LEDD suggested by each rater. We then evaluated the agreement between raters about the direction of the total LEDD modification (increase, decrease or stabilization; range [50; 100]%).

A correlation study was performed between the interrater agreements and the integrated PKG parameters using Pearson’s method (normality validated with the Shapiro–Wilk test). Spearman’s correlation method was used to study the relationship between the interrater agreements and the continuous PKG parameters.

We performed a 3-class-one-versus-all logistic regression after bootstrapping the 64 combinations (8 cases and 8 raters) to describe and estimate the impact of the patient’s case per se (case variable), the rater, the patient’s age, the integrated PKG scores summarized as FDS, PTI and PTT, on the direction of the total LEDD modification. Thus, the three logistic regressions show the tendency of the previously listed factors to act on the increase or not of the total LEDD, to act on its stabilization or not, and to act on its decrease or not. The results are presented as the coefficients of each of the logistic regressions to evaluate the decision that was the most influenced by the studied factors. Because amantadine could be added in order to reduce dyskinesia, a LEDD increase only due to amantadine adjunction was classified as equivalent to a treatment decrease^30^.

We used Python Software Foundation to perform the analysis (Python Language Reference, version 3.7.4 Available at http://www.python.org).

### Reporting summary

Further information on research design is available in the Nature Research Reporting Summary linked to this article.

## Supplementary information

Supplementary Material

Reporting Summary

## Data Availability

Data are available upon request from the corresponding author at the Department of Neurology of the Salpêtrière Hospital (Paris, France).
