# Supplementary material for: Symptoms assessment and decision to treat patients with advanced Parkinson’s disease based on wearables data
Source: NPJ Parkinsons Dis. 2023 Mar 27;9:45. doi: 10.1038/s41531-023-00489-x (PMC10042860; doi:10.1038/s41531-023-00489-x)
Supplement: Supplementary file 1 — Supplementary Material [file 41531_2023_489_MOESM1_ESM.pdf]

Supplementary Table 1: Percentages of agreement between raters for each symptom analyzed and treatment adaptation decisions per patient's case

**Patients' Cases**	**n°1**	**n°2**	**n°3**	**n°4**	**n°5**	**n°6**	**n°7**	**n°8**
**Agreement on BK** (%)	71.7	93.4	98.9	82.7	100.0	77.9	84.6	94.5
**Agreement on DK** (%)	80.1	84.2	93.8	100.0	71.3	91.9	100.0	95.6
**Agreement on tremor** (%)	92.6	100.0	100.0	89.0	99.3	100.0	89.0	97.8
**Agreement on treatment adaptation direction** (%)	87.5	62.5	75	87.5	100	75	100	50

Supplementary Table 2: Impacts of the patient's case and the rater on the treatment modification: increase, stabilization or decrease of total LEDD

	**Increase** (n= 29)		**Stabilization** (n= 8)		**Decrease** (n= 27)	
	mean	*ic90%*	*mean*	*ic90%*	*mean*	*ic90%*
**Patients' Cases**						
n°1	0.474	0.260, 0.768	-0.406	-0.491, -0.288	-0.358	-0.649, -0.155
n°2	0.258	-0.087, 0.534	-0.705	-0.835, -0.533	0.055	-0.227, 0.375
n°3	-0.839	-0.969, -0.671	2.101	1.838, 2.411	-0.599	-0.890, -0.327
n°4	0.720	0.569, 0.855	-0.592	-0.694, -0.449	-0.553	-0.665, -0.421
n°5	-0.773	-0.912, -0.603	-0.609	-0.724, -0.447	1.067	0.869, 1.248
n°6	-0.331	-0.626, -0.125	-0.684	-0.797, -0.531	0.683	0.448, 0.972
n°7	0.768	0.624, 0.900	-0.379	-0.459, -0.278	-0.609	-0.723, -0.473
n°8	-0.416	-0.726, -0.120	0.740	0.314, 1.121	0.121	-0.194, 0.470
**Raters**						
neuro1	-0.224	-0.412, -0.030	0.637	0.081, 1.014	-0.157	-0.420, 0.114
neuro2	-0.229	-0.417, -0.032	-0.067	-0.520, 0.225	0.205	-0.066, 0.459
neuro3	0.110	-0.204, 0.365	0.630	0.044, 1.017	-0.502	-0.760, -0.218
neuro4	0.451	0.113, 0.765	-0.076	-0.515, 0.227	-0.520	-0.879, -0.168
neuro5	0.121	-0.189, 0.359	-0.766	-0.932, -0.409	0.201	-0.103, 0.512
neuro6	0.473	0.174, 0.733	-0.066	-0.521, 0.221	-0.510	-0.748, -0.244
neuro7	-0.611	-0.841, -0.268	-0.068	-0.521, 0.231	0.532	0.198, 0.839
neuro8	-0.230	-0.420, -0.020	-0.760	-0.927, -0.419	0.558	0.267, 0.807

mean: estimated effect of the factor from the 1000 iterative resamplings at 80% of the 64 combinations

ic90%: credible interval at 90% from the distribution of the bootstrapping distribution
